# RETRACTION: Uroprotective and Hepatoprotective Potential of *Anagallis Arvensis* Against the Experimental Animal Model

**DOI:** 10.1155/jotm/9817092

**Published:** 2026-07-29

**Authors:** Journal of Tropical Medicine

RETRACTION: U. Shabbir, I. Anjum, M. Naveed Mushtaq, et al., “Uroprotective and Hepatoprotective Potential of *Anagallis Arvensis* Against the Experimental Animal Model,” *Journal of Tropical Medicine* (2022): https://doi.org/10.1155/2022/7241121.

The above article, published online on 26 September 2022 in Wiley Online Library (https://wileyonlinelibrary.com), has been retracted by agreement between the *Journal of Tropical Medicine*; and John Wiley & Sons Ltd. The retraction has been agreed due to concerns raised by third parties. Specifically, Figure 3c was found to have been previously published by the same author group in a different scientific context (DOI:10.1021/acsomega.1c07292). Although the authors acknowledged the error and supplied materials to correct it, further investigation by the publisher has identified the presence of irrelevant and only marginally relevant references, resulting in inadequate support from the literature and an insufficiently justified study rationale. Finally, some of the reported results were found not supported by the presented data. Accordingly, the article has been retracted. The corresponding author, Irfan Anjum, disagreed with the retraction decision. No confirmation was received from the remaining co‐authors.

